# Knowledge and Attitude of Healthcare Practitioners Toward Therapeutic Drug Monitoring Practices in the Najran Region, Kingdom of Saudi Arabia

**DOI:** 10.7759/cureus.32214

**Published:** 2022-12-05

**Authors:** Mohammad Al Mutarid, Abdulaziz Alhossan, Tanveer Khan, Mana G Alyami, Koblan M Almutared, Mohammed Alshiban, Ali Hamad D Alyami, Mutared Mohammed M Alyami, Jaamil Ali H AlKulayb, Daji S Alyami, Qublan D Almutarid

**Affiliations:** 1 Pharmaceutical Care, General Hospital, Najran, Ministry of Health Saudi Arabia, Najran, SAU; 2 College of Pharmacy, King Saud University, Riyadh, SAU; 3 College of Pharmacy, Riyadh Elm University, Riyadh, SAU; 4 Administration of Quality, Ministry of Health, Najran, SAU; 5 Administration of Pharmaceutical Care, Ministry of Health, Najran, SAU; 6 Planning and Development Department, Ministry of Health, Najran, SAU; 7 Pharmaceutical Care Services, Maternity and Children Hospital, Ministry of Health, Najran, SAU; 8 Pharmaceutical Care Services, Khobash General Hospital, Najran, SAU

**Keywords:** saudi arabia, attitude of health personnel, knowledge level, healthcare provider, therapeutic drug monitoring

## Abstract

Background: Therapeutic drug monitoring (TDM) is one of the useful clinical tools that aim to improve and ensure the best therapeutic effects while avoiding drug-related toxicity. We aimed to evaluate the knowledge and attitude toward TDM practices among healthcare practitioners in the Najran region, Saudi Arabia.

Methodology: A cross-sectional study was conducted between May 2021 and October 2021 for assessing the awareness and attitude of licensed doctors and pharmacists working in Saudi Arabia regarding TDM practices. A 31-item questionnaire was distributed to the healthcare professionals via an electronic link. Descriptive and inferential statistics were used to report the data.

Results: A total of 392 participants submitted questionnaires. More than half of the professionals (55.9%) had an overall awareness of the TDM, while only 3.1% did not. Only 16% and 23% of those who were surveyed indicated that TDM is used at the beginning of medication administration and when shifting from one drug to another, respectively. The majority of the professionals responded that TDM is revealed with laboratory changes in liver and kidney function (81%), and TDM is specified with suspected therapeutic failure (93%). Only half of the respondents claimed that they had ever requested or suggested a TDM in their practice. More than 90% of the respondents claimed that they were aware of the indications for vancomycin (n = 381), gentamicin (n = 375), lithium (n = 369), digoxin (n = 380), and theophylline (n = 369).

Conclusion: This study found that the majority of the healthcare professionals in Najran were aware of the TDM, particularly those with more than 10 years of professional experience. In addition, TDM service is not widely available in smaller hospitals in Najran. There is a need to conduct customized training programs for junior healthcare professionals, thus increasing the levels of awareness toward TDM. Future studies in Saudi Arabia can explore the role of clinical pharmacists in the provision of TDM services by assessing the clinical and economic outcomes of pharmacist interventions in TDM.

## Introduction

Therapeutic drug monitoring (TDM) is defined as “measuring serum concentrations of a drug at single or multiple time points in a biological matrix after a dosage with appropriate medical interpretation [1].” The main objective of TDM is to personalize the dosage to attain maximum drug efficacy and at the same time reduce the occurrence of drug-related problems [2]. TDM has clinical importance for medications with a narrow therapeutic index such as phenytoin, digoxin, methotrexate, and gentamycin [3]. Altered pharmacokinetic parameters may deliver appropriate and safe medication therapy through maintaining concentrations of drug in the blood within a therapeutic range or window (maximum therapeutic concentrations with a minimum toxic concentration) [4]. The therapeutic range has usually been described for several medications in disease situations such as cardiovascular disease, thyroid dysfunction, renal and hepatic impairment, and cystic fibrosis. For pregnant women, altered drug disposition similarly occurs. TDM assists in identifying altered drug dispositions and the adjustment of dosage can be applied for effective management of the patient to evade adverse drug reactions and cost-effective in health care [2].

In the 1970s, TDM concentrated on drug safety, and reduced the occurrence of toxicity through determination and confirmed therapeutic ranges [5]. After this, the clinical pharmacokinetics of TDM increased focusing on drug pharmacokinetic characteristics, drug concentration relationships, and developments in analytical technology. Today TDM comprises monitoring of patient compliance, drug efficiency, drug-drug interactions, drug-food interaction and individual variation in therapy response, adverse effects, and therapy cessation [1]. Frequent guidelines have been established that contain sampling time policies, accurate indications, and correct utilization of serum drug concentrations [6].

Drug monitoring facilities became one of the most important subjects in modern hospitals between clinicians, clinical pharmacists, and pharmacologists in the former two decades. The enhanced awareness about the association between drug concentration and therapeutic response among several physicians in different specialties worldwide encouraged a wide range of computerized analytical technology involved in TDM [7]. Healthcare practitioners interpreted the results and selected the proper drug and adequate dosage as per the patient’s needs. Healthcare practitioners used TDM as a treatment guideline and got an excellent pharmacological response with fewer adverse toxic effects [8]. By combining pharmaceutical knowledge of pharmacokinetics, pharmacodynamics, and pharmaceutics, TDM enables the evaluation of the efficacy and safety of a specific medicine in a variety of clinical settings [5,9]. TDM depended on aware monitoring of both pharmacodynamics and pharmacokinetics to earn the greatest therapeutic benefit of any medical drug within the minimal dosage with the least expected adverse effects and toxicities, especially in vulnerable groups such as cardiac, hepatic, and renal patients in addition to pediatrics and pregnant women [10]. 

In Saudi Arabia, the TDM service was formerly recognized, but this service is still limited, especially in decentralized regions [11]. The application of TDM in Saudi Arabia is being improved each year to include more drugs, and the number of TDM requests is increasing [12]. Recently, numerous Ministry of Health hospitals have been delivering TDM services as part of their routine drug monitoring in patient management. The increase in the availability of TDM services in Saudi Arabia is probably due to increasing awareness of its clinical benefits and advanced automated analytical techniques [13]. Najran is a region on the border with Yemen in the southern part of Saudi Arabia. Governmental healthcare services are provided in the Najran region through ten general hospitals. TDM service is provided through a central well-equipped regional laboratory through numerous healthcare practitioners. Evidence indicates that there is limited data regarding TDM awareness among healthcare professionals working in the Najran region. Therefore, we aimed to evaluate the knowledge and attitude toward TDM practices among healthcare practitioners in the Najran region, Saudi Arabia, and factors associated with a higher number of reported medications indicating TDM.

## Materials and methods

We conducted a cross-sectional study between May 2021 and October 2021 for assessing the awareness and attitude of licensed doctors and pharmacists (working in the Najran region of the Kingdom of Saudi Arabia) regarding TDM practices.

A 31-item questionnaire (Appendix 1, Table 5) was designed to explore the awareness and attitude of healthcare professionals regarding TDM. The self-administered questionnaire was adapted from previous studies [14]. The questionnaire comprised questions on the following domains: general information about participants, awareness, and attitude of healthcare professionals toward TDM, and barriers to not using TDM in hospitals. A web link to the survey questionnaire was distributed to licensed physicians and pharmacists working in the Najran-based government hospitals. A convenient sampling strategy was followed. The objectives of this research project were also included on the front page of the study questionnaire under the consent statement to deliver general information to the study participants. This research study was commenced after receiving approval from the Institutional Review Boards (IRBs) of Riyadh Elm University (IRB approval number: FPGRP/2021/584/502/491) and the Ministry of Health (IRB approval number: 2021-31E).

A total of 1400 physicians and pharmacists work in Najran. To elicit a response from 50% of respondents, with a 95% confidence level and a 5% margin of error, a sample size of 302 was estimated. An online sample size calculator was used in determining the minimum sample size for this study [15]. Chi-square and Fisher exact tests were used to compare the awareness scores among the different participant groups whenever appropriate. Statistical analyses were conducted by using the R software version. 4.0.5 (R Studio: Integrated Development for R, Boston, MA). Logistic regression was applied to identify the risk factors associated with the high number of reported drugs indicating TDM. Significance was deemed with a p-value less than 0.05.

## Results

Table 1 provides a summary of the demographic characteristics of the study participants. Three hundred ninety-two healthcare practitioners participated in the survey. Of these, 54% of the practitioners were female. Over 70% of the practitioners were under 45 years old. Over half the practitioners (55%) were working in a hospital with a bed size of 50 or less. Over 75% of those surveyed were either medical residents or pharmacists. About 40% of the respondents had more than 10 years of job experience.

**Table 1:** Demographic characteristics of the healthcare professionals. *Private program (n = 2), epidemiology inspector (n = 1), technician (n = 2), nurse (n = 2), social worker (n = 1), and engineering (n = 1).

Characteristic	n (%)
Age (in years)	
25–34	115 (29%)
35–44	174 (44%)
45–54	88 (22%)
55 and above	15 (3.8%)
Sex	
Female	212 (54%)
Male	180 (46%)
Hospital’s bed size	
<50 Beds	214 (55%)
>50 Beds	178 (45%)
Job type	
Medical consultant	18 (4.6%)
Medical specialist	49 (12%)
Medical resident	158 (40%)
Consultant pharmacist	5 (1.3%)
Senior pharmacist	11 (2.8%)
Pharmacist	142 (36%)
Other*	9 (2.3%)
Job experience (in years)	
<1	9 (2.3%)
1–4	48 (12%)
5–9	179 (46%)
10–14	124 (32%)
>15	32 (8.2%)

More than half of the professionals (55.9%) had an overall awareness of the TDM, while only 3% did not demonstrate awareness of the TDM (Table 2). There were statistically significant differences (p < 0.001) between the age, gender, and job experience of the healthcare professionals and awareness of the TDM. Unlike the job type, the hospital’s bed size, where the professionals were working, also had an influence on their awareness of the TDM.

**Table 2:** Healthcare professionals’ awareness of therapeutic drug monitoring. TDM: therapeutic drug monitoring. p-values: Fisher's exact test; Pearson's Chi-squared test.

Variable	Overall awareness, n (%)			Correct responses for the indication of TDM, n (%)			
	No, N = 12	Partially, N = 161	Yes, N = 219	Liver and/or kidney impairment, N = 319	Shift from a medication to another, N = 90	Suspected therapeutic failure, N = 365	Onset of treatment, N = 62
Age (years)	p < 0.001			p = 0.572	p = 0.003	p = 0.071	p < 0.001
25–34	6 (50%)	66 (41%)	43 (20%)	90 (28%)	31 (34%)	102 (28%)	18 (29%)
35–44	3 (25%)	78 (48%)	93 (42%)	143 (45%)	48 (53%)	164 (45%)	40 (65%)
45 and above	3 (25%)	17 (11%)	83 (38%)	86 (27%)	11 (12%)	99 (27%)	4 (6.5%)
Sex	p < 0.001			p = 0.512	p < 0.001	p < 0.001	p < 0.001
Female	4 (33%)	108 (67%)	100 (46%)	170 (53%)	23 (26%)	206 (56%)	8 (13%)
Male	8 (67%)	53 (33%)	119 (54%)	149 (47%)	67 (74%)	159 (44%)	54 (87%)
Hospital’s bed size	p < 0.001			p = 0.109	p = 0.098	p = 0.767	p = 0.749
50 Beds or less	8 (67%)	133 (83%)	73 (33%)	168 (53%)	56 (62%)	200 (55%)	35 (56%)
More than 50 beds	4 (33%)	28 (17%)	146 (67%)	151 (47%)	34 (38%)	165 (45%)	27 (44%)
Job type	p = 0.128			p = 0.748	p < 0.001	p = 0.012	p < 0.001
Medical professionals	7 (58%)	88 (55%)	130 (59%)	185 (58%)	41 (46%)	214 (59%)	22 (35%)
Pharmacy professionals	4 (33%)	72 (45%)	82 (37%)	126 (39%)	41 (46%)	145 (40%)	33 (53%)
Others	1 (8.3%)	1 (0.6%)	7 (3.2%)	8 (2.5%)	8 (8.9%)	6 (1.6%)	7 (11%)
Job experience (in years)	p < 0.001			p = 0.473	p = 0.031	p = 0.563	p < 0.001
<5	2 (17%)	35 (22%)	20 (9.1%)	48 (15%)	20 (22%)	52 (14%)	18 (29%)
5–9	7 (58%)	93 (58%)	79 (36%)	141 (44%)	33 (37%)	169 (46%)	14 (23%)
>10	3 (25%)	33 (20%)	120 (55%)	130 (41%)	37 (41%)	144 (39%)	30 (48%)

Only 16% and 23% of those who were surveyed indicated that TDM was used at the beginning of medication administration and when shifting from one drug to another, respectively. On the other hand, most of the professionals responded that TDM is revealed with laboratory changes in liver and kidney function (81%), and TDM is specified with suspected therapeutic failure (93%). None of the professionals’ characteristics had been associated with their awareness of TDM’s indication during the liver and/or kidney impairment. Except for the working site (i.e., bed size), there were statistically significant differences between the other demographic variables and awareness of the shift from one medication to another and the onset of treatment (Table 2).

Only half of the respondents claimed that they had ever requested or suggested a TDM in their practice. More than 90% of the respondents claimed that they were aware of the indications for vancomycin, gentamicin, antiepileptics, lithium, digoxin, and theophylline, which are targeted by TDM assays (Table 3).

**Table 3:** Awareness of indications for requesting TDM assays and drugs targeted by TDM assays. TDM: therapeutic drug monitoring.

Awareness	N (%)
TDM is revealed with laboratory changes in liver and kidney function	319 (81%)
TDM is specified with suspected therapeutic failure	365 (93%)
TDM is used at the beginning of medication administration	62 (16%)
TDM is used when shifting from a drug to another	90 (23%)
Have you ever requested/suggested a TDM in your practice?	196 (50%)
Antibiotics requiring TDM	
Tobramycin	325 (83%)
Vancomycin	381 (97%)
Gentamicin	375 (96%)
Antiepileptics requiring TDM	
Carbamazepine	379 (97%)
Phenobarbital	376 (96%)
Phenytoin	381 (97%)
Valproic acid	375 (96%)
Antipsychotics/antidepressants requiring TDM	
Imipramine	257 (66%)
Lithium	369 (94%)
Clozapine	299 (76%)
Antiarrhythmics requiring TDM	
Digoxin	380 (97%)
Disopyramide	78 (20%)
Lidocaine	55 (14%)
Propranolol	46 (12%)
Quinidine	272 (69%)
Others requiring TDM	
Theophylline	369 (94%)
Methotrexate	373 (95%)
Acetaminophen	68 (17%)
Salicylates	46 (12%)

After adjustments for other factors (Table 4), only the working site was significantly associated with a higher number of reported medications indicating TDM. Professionals working in hospitals with more than 50-bed capacity were twice more to report a higher number of medications indicating TDM (adjusted odds ratio (AOR): 2.25, 95% CI: 1.38-3.72, p = 0.001) than those working in hospitals with 50-bed or less occupancy.

**Table 4:** Factors associated with a higher number of reported medications indicating TDM. AOR: adjusted odds ratio, CI: confidence interval, TDM: therapeutic drug monitoring. ^a^n (%), ^b^Pearson's Chi-squared test; Fisher's exact test, *median was taken as a cut-off point.

Variable	Number of reported medications indicating TDM*			Multivariate logistic regression	
	<13 (N = 121)^a^	≥13 (N = 271)^a^	P-value^b^	AOR (95% CI)	P-value
Age (years)			0.004		
25–34	48 (40%)	67 (25%)		1.00	—
35–44	51 (42%)	123 (45%)		1.49 (0.80–2.77)	0.2
45 and above	22 (18%)	81 (30%)		1.82 (0.75 – 4.47)	0.2
Sex			0.923		
Female	65 (54%)	147 (54%)		1.00	—
Male	56 (46%)	124 (46%)		1.04 (0.65–1.66)	0.9
Hospital’s bed size			<0.001		
50 Beds or less	85 (70%)	129 (48%)		1.00	—
More than 50 beds	36 (30%)	142 (52%)		2.25 (1.38–3.72)	0.001
Job type			0.003		
Medical professionals	55 (45%)	170 (63%)		1.00	—
Pharmacy professionals	64 (53%)	94 (35%)		0.60 (0.36–1.01)	0.053
Others	2 (1.7%)	7 (2.6%)		1.49 (0.31–10.9)	0.6
Job experience (in years)			0.189		
Hospital’s bed size	22 (18%)	35 (13%)		1.00	—
50 Beds or less	58 (48%)	121 (45%)		0.85 (0.41–1.73)	0.7
More than 50 beds	41 (34%)	115 (42%)		0.73 (0.30–1.76)	0.5

**Figure 1:**
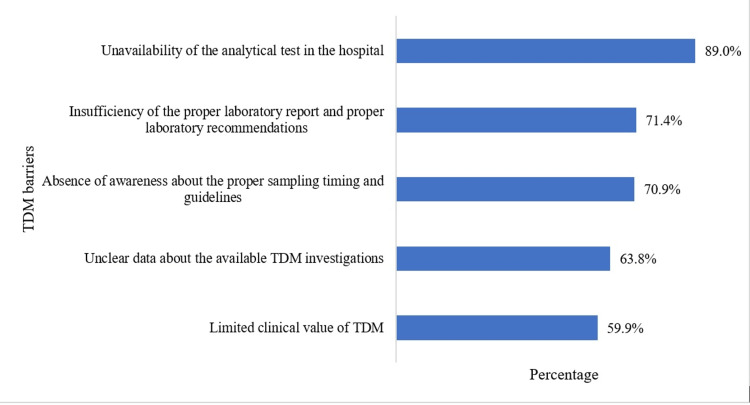
Barriers for not using TDM in the hospital TDM: therapeutic drug monitoring.

Respondents were also asked to mention barriers to not using TDM in their hospitals (Figure 1). The most prominent barrier mentioned was the unavailability of the analytical test in the hospital (n = 349, 89.0%), followed by the insufficiency of the proper laboratory report and proper laboratory recommendations (n = 280, 71.4%) and the absence of awareness about the proper sampling timing and guidelines (n = 278, 70.9%).

## Discussion

This study was performed using an anonymous electronic questionnaire to assess the level of awareness and attitude of Najran-based healthcare professionals, such as doctors and pharmacists, toward TDM practices. World Health Organization highlights that there is a lack of an appropriate setting of a system of surveillance for drug-drug interactions, irrational prescribing, and polypharmacy [16]. These problems demonstrate many underlying systematic loops. Healthcare professionals are overburdened, and disproportionality between patients and resources adds to these concerns [16].

The majority of the healthcare professionals in our study had an overall awareness of the TDM, which is consistent with that of other studies conducted among healthcare professionals [14,16,17]. Similarly, this study found that the bed size of the hospital influenced the awareness of the TDM. Healthcare professionals, particularly medical residents from the hospital with bed sizes less than 50, mostly responded to the study questionnaire. Regarding the awareness of the healthcare professionals about the indication of TDM, suspected therapeutic failure, and altered liver or kidney functions were the most common indications known by these professionals who participated in the study. These findings are also in line with the previous study [14]. Awareness about the medications for TDM and the general indications of TDM was significantly higher among healthcare professionals with more than 10 years of experience in the medical practice.

Regarding the barriers that limit the application of TDM in Najran according to the opinion of healthcare professionals, study participants reported that the unavailability of the analytical test in the hospital was the most important barrier to not using TDM in the hospital, followed by the insufficiency of the proper laboratory report and proper laboratory recommendations and the absence of awareness about the proper sampling timing and guidelines. The previous study also reported the insufficiency of a proper laboratory report with guiding recommendations [14]. For drugs with a narrow therapeutic index, it is crucial to ask the laboratory of the hospital for details on the proper sampling technique and post-dosage timing [18]. The finding related to the unavailability of the analytical test in many hospitals in Najran contradicts the findings of the Malaysian study, where TDM service is generally available in government hospitals [19]. Around two-thirds of Malaysian hospitals provide service with their internal TDM laboratories [19].

Half of the healthcare professionals in our study claimed that they had ever requested or suggested a TDM in their practice and were aware of the indications for vancomycin, gentamicin, antiepileptics, lithium, digoxin, and theophylline, which are targeted by TDM assays. This evidence is consistent with those of other reports [14,20].

In this study, the size of the hospital showed a significant association with a higher number of reported medications, indicating TDM. Healthcare professionals working in hospitals with more than a 50-bed capacity were twice more to report a higher number of medications indicating TDM than those working in hospitals with a 50-bed or less occupancy. In our study, most of the healthcare professionals work in a small hospital with less than 50 beds, which is also one of the reasons for not providing TDM services. Likewise, a previous study in Malaysia reported that hospitals with a smaller number of beds lack TDM service [19]. Another study reported that not all hospitals in Australia provide TDM services in their own laboratories [21]. Hospitals with a smaller number of beds generally depend on bigger hospitals to render the TDM service, where samples of patients are sent out for drug assay [19]. In hospitals in developed countries, TDM service is provided 24 hours a day [22,23].

The most important strength of this study was the diversity among healthcare professionals who responded to the study questionnaire from different areas of Najran. However, there are certain limitations as well. The participants only belonged to one state. The nature of this study meant that we superficially explored this research area, and more detailed work would be of value in the future, including exploring the reasons for the unavailability of TDM service in smaller hospitals nationally and strategies to overcome barriers. Another limitation is related to the self-reported questionnaire, as outcomes may certainly be influenced by healthcare professionals’ subjective factors. However, it is anticipated that the anonymity of the study questionnaire urged honesty.

## Conclusions

This study found that majority of the healthcare professionals in Najran were well aware of the TDM, particularly those with more than 10 years of professional experience. In addition, TDM service is not widely available in smaller hospitals in Najran. There is a need to conduct customized training programs for junior healthcare professionals, thus increasing the levels of awareness toward TDM. Future studies in Saudi Arabia can explore the role of clinical pharmacists in the provision of TDM services by assessing the clinical and economic outcomes of pharmacist intervention in TDM, and their knowledge, attitude, and practices toward TDM.

## Appendices

Appendix 1

**Table 5:** Questionnaire TDM: therapeutic drug monitoring.

31-Item questionnaire	
Age (in years)	25–34
	35–44
	45–54
	55 and above
Sex	Female
	Male
Hospital’s bed size	<50 Beds
	>50 Beds
Job type	Medical consultant
	Medical specialist
	Medical resident
	Consultant pharmacist
	Senior pharmacist
	Pharmacist
	Other
Job experience (in years)	<1
	1–4
	5–9
	10–14
	>15
Are you aware of therapeutic drug monitoring (TDM) concept?	Yes, No, Not sure
TDM is revealed with laboratory changes in liver and kidney function	True, False
TDM is specified with suspected therapeutic failure	True, False
TDM is used at the beginning of medication administration	True, False
TDM is used when shifting from a drug to another	True, False
Have you ever requested/suggested a TDM in your practice?	Yes, No
Tobramycin requires TDM	True, False
Vancomycin requires TDM	True, False
Gentamicin requires TDM	True, False
Carbamazepine requires TDM	True, False
Phenobarbital requires TDM	True, False
Phenytoin requires TDM	True, False
Valproic acid requires TDM	True, False
Imipramine requires TDM	True, False
Lithium requires TDM	True, False
Clozapine requires TDM	True, False
Digoxin requires TDM	True, False
Disopyramide requires TDM	True, False
Lidocaine requires TDM	True, False
Propranolol requires TDM	True, False
Quinidine requires TDM	True, False
Theophylline requires TDM	True, False
Methotrexate requires TDM	True, False
Acetaminophen requires TDM	True, False
Salicylates requires TDM	True, False
